# Mobile health intervention for promotion of eye health literacy

**DOI:** 10.1371/journal.pgph.0000025

**Published:** 2021-10-13

**Authors:** Indra Prasad Sharma, Monica Chaudhry, Dhanapati Sharma, Raju Kaiti

**Affiliations:** 1 GKCW National Eye Center, JDW National Referral Hospital, Thimphu, Bhutan; 2 Department of Optometry and Vision Science, Amity University Haryana, Gurugram, India; 3 Department of English, Gedu College of Business Studies, Chukha, Bhutan; 4 Department of Optometry, Nepal Eye Hospital, Tripureshwor, Nepal; Anglia Ruskin University - Cambridge Campus, UNITED KINGDOM

## Abstract

**Purpose:**

Improving eye health awareness in the underserved population is a universal eye health priority. The ubiquity of cell phones and internet usage provides new and innovative opportunities for health promotion. This study evaluated the effect of mobile health intervention (text message link) to promote eye health literacy (EHL) of priority ocular morbidities.

**Methods:**

This study was an intervention evaluation and employed a two-armed pre-test post-test approach. Baseline assessment on EHL was performed on 424 university students. Participants were categorised into intervention and control groups, using the 1:1 allocation ratio. The intervention and control group received a text message alone and text message with a link, respectively. EHL was assessed via a self-administered questionnaire. The primary outcome measures were changes in EHL scores between baseline and one month post-intervention. Descriptive analysis was performed to assess the cost-effectiveness of the intervention.

**Results:**

With low attrition and a response rate of 95.6%, 409 responses were eligible for analysis. The mean age of the participants (49.4% males and 50.6% of females) was 19.9±1.68 years. Baseline EHL scores were low, and there was no correlation with a demographic profile (all p<0.05, CI 95%). The demographic characteristics were similar between the two groups (for all, P <0.05, CI 95%) at baseline. The EHL scores improved in both groups between the pre-and post-test assessment; however, improvements were statistically significant only in the control group. The one-month post-intervention EHL scores were also higher in the intervention group compared to the control (p≤0.001, CI 95% for all). The total cost incurred for the intervention used was 11.5 USD.

**Conclusion:**

Text message link demonstrated effectiveness for improving the EHL scores; the low baseline EHL scores substantially improved with intervention. The text message link intervention is a cost-effective method and could be considered in advocating for eye health in developing countries, particularly during global emergencies.

## Introduction

Global eye health is an emerging public health challenge of the 21st century. With the global population growing and aging, more people are developing and living with visual impairment (VI) [1]. VI poses a tremendous socioeconomic burden and affects the quality of life, ultimately plunging individuals into the vicious circle of poverty [2, 3]. The eye health progress is not keeping pace with needs, and we continue to face enormous challenges in elimination avoidable VI [4]. The World Report on Vision (WRV 2019) estimated that at least 2.2 billion people have VI, of which almost half could be prevented or has yet to be addressed. Uncorrected refractive error (43%) followed by unoperated cataract (33%), glaucoma (2%), and diabetic retinopathy (1%) are the leading causes of VI and are considered the priority ocular morbidities [5]. Literature suggests that with raised eye health awareness and provision of primary eye care services, 4/5th of VI are avoidable [5].

To improve universal eye health, the WRV recommends raising eye health awareness, engaging communities and empowering people about eye care needs. Literature acknowledges that the awareness and knowledge of common ocular morbidities are poor among the general population, causing a major barrier to uptake of eye care services [6–9]. The Low middle-income countries (LMICs) face ophthalmic human resources and financial constraints; providing eye health services and information to the population is a confronting task. Screening camps and awareness programs must meet to provide advocacy [10]. Elevating eye health literacy (EHL) within the key audiences plays a paramount role in eliminating avoidable blindness. During this COVID-19 pandemic, the need to identify and utilize cost-effective public health intervention is felt more than ever. In the LMICs, traditional ways of health education are time and resource-consuming and are least workable during pandemics. It calls for exploring the effectiveness of mobile health interventions that could aid health policymakers in planning interventions.

eHealth Technologies have emerged as an inexpensive, fast, and dynamic method of disseminating health information [11]. Besides with vast penetration and widespread reach, text messages with reliable links make it suitable for public health practices. However, with merely a few studies conducted to evaluate the effectiveness of text messages in health advocacy, the tool remains underutilized by public health professionals and policymakers [12–16]. Furthermore, eye health promotion has not received adequate priority [17]. With the ubiquity of mobile phone usage in a diverse population, mobile health interventions appear to be a promising medium for improving health education for all ages [18–20].

Considering the proliferation of mobile phones, and easy internet access amongst a diverse population, the study aimed to assess the effect of mobile health intervention (text message link) to promote EHL of priority eye diseases among university students. The hypotheses were; (1) the intervention group will have a higher EHL score after mobile health intervention (text message link) than of baseline, and (2) the intervention group will have a higher post intervention EHL score compared to the control group.

## Methods

### Design

This two-armed, parallel group (1:1) pre-test post-test questionnaire-based study evaluated the effect of mobile health intervention (text message link). Prior to commencement, study design and protocol got approved by the Institutional Ethical Committee (IRC) of the Amity University Haryana (AUH), Haryana, India (Reference: AUH/EC/D/2016/31). The study adhered to the tenets of the Declaration of Helsinki for human participants and followed the principles of Good Clinical Practice (GCP).

### Participants

Eligible participants were; (1) university students and (2) aged above 18 years. The study enrolled participants owning a smartphone with an active phone number and anticipating participation throughout the study. To reduce study bias, enrollment excluded students pursuing courses in optometry and visual sciences. Participants were invited through the university website and recruited between January and March 2018. It was implemented in three stages: baseline assessment, allocation and intervention, and post-intervention evaluation within six months (January to July 2018). On completing the study, participants received a free comprehensive eye examination at the Amity Optometry Clinic.

### Procedure

Data collectors attended a one-day training session covering necessary skills in data collection techniques, confidentiality, and privacy assurance. Participants fulfilling eligibility criteria were enrolled, informed that they were recruited for an interventional study and asked to sign an informed consent. Each participant was assigned a unique code to mask personal identifiers. Following enrollment, the socio-demographic information and web-enabled personal mobile phone number were recorded. Prior to randomisation and allocation, a EHL baseline assessment (pre-test) was conducted using a self-administered questionnaire. The intervention was assigned and the post-intervention assessment was conducted for both groups on the 30th day after the baseline assessment.

### Development of study questionnaire

A structured questionnaire was developed in English by the investigators [**S1 File]**. It was designed to assess the EHL (total scores on awareness and knowledge) of cataract, diabetic retinopathy, glaucoma, and refractive error. The questionnaire’s face and content validity were assessed by faculties and reviewed by an expert panel of the university. The reproducibility and validity of the questionnaire were verified through a pilot study comprising 10% (n = 40) of the study population. The experience and feedback received from the pilot study were used to resolve the discrepancies and refine the questionnaire.

#### Awareness

The first question, which evaluated the individual’s awareness, comprised whether the respondent had ever heard the name of the disease. Close-ended responses (yes or no) were recorded. Scores for yes and no were recorded as 1 and 0, respectively.

#### Knowledge

Responses to open-ended questions on symptoms and treatment options for each ocular condition were assessed. Providing at least one simple and correct symptom and treatment option of the disease was considered having knowledge; correct as 1 and incorrect as 0. Overall knowledge score was the total of scores on knowledge of symptoms and treatment options. The correct and incorrect responses participants provided are documented in **S1 Table**.

### Intervention

**Fig 1:**
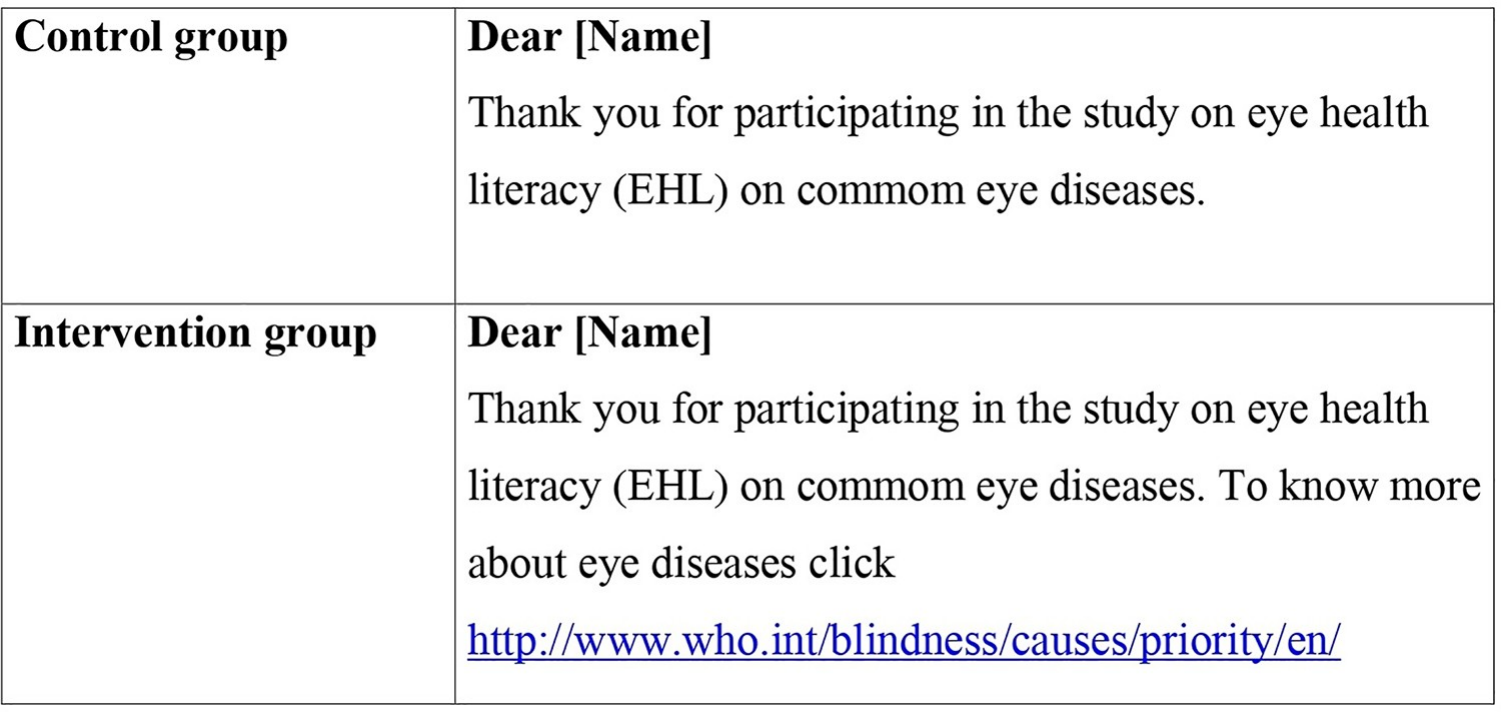
Tailored text message for intervention and control group.

The intervention was a mobile health intervention (text message link) delivered as a text message. Two short text messages (SMS) were tailored; (1) a text message thanking participants for taking part in the baseline study (control text message) and, (2) a text message thanking participants for taking part in the baseline study along with a hyperlink (http://www.who.int/blindness/causes/priority/en/ ) (intervention text message) **[Fig 1].** The hyperlink opened the WHO website on priority eye diseases containing a brief description of the background, causes, common symptoms, and treatment options for the priority eye diseases; cataract, glaucoma, diabetic retinopathy, and refractive error. The WHO website was chosen to provide reliable and consistent information about eye diseases. Participants allocated to control and intervention groups received the control text message and intervention text message, respectively, on the fifth day of baseline assessment.

### Outcome measures

All outcomes were self-reported and collected through a survey. The primary outcome measures included: (1) awareness, (2) knowledge, (3) changes in EHL (awareness and knowledge) scores for cataract, glaucoma, diabetic retinopathy, and refractive error from baseline to one-month post-test. Having heard about the disease was considered as having awareness and demonstrating some understanding about the symptoms and treatment options was considered having knowledge. The responses received from the participants are shown in S1 Table. The secondary outcome denoted the assessment of the cost-effectiveness of the intervention during the study period (presented separately).

### Sampling and sample size

An *a priori* power calculation was conducted to determine the sample size required. Assuming an effect size of 0.04 (small) between the two groups, and an alpha error of 0.05 (two-tailed), it required 328 participants to give power (1 - β) of over 95%. Considering a follow-up rate of 80%, and attrition at 15%, 442 questionnaires were distributed during the pre-intervention test. The sampling frame used student enrollment numbers from the university registry and participants selected by random selection, generated using Microsoft Excel.

### Randomization and blinding

After the baseline assessment (pre-test), the randomization was performed by the statistician. A list of participant numbers (unique to this study) was prepared, and computer-generated randomization allocated the participants to intervention and control groups in a 1:1 allocation sequence. The allocation was concealed from the participants, study staff, and investigators until the intervention was assigned. The data were collected by trained optometrists and analyzed by a statistician; both blinded to the intervention throughout the study. The participants and investigators delivering intervention could not be blinded due to the nature of the study.

### Data management and statistical analysis

Data coding, quality control, and data entry were done using established procedures. The questionnaires were pre-coded to minimize data coding errors. Before data entry, forms were checked for errors and necessary corrections made. Data were double entered by two different investigators using Epi-data version 3.1. Tools and checks of Epi-data software were used to control data entry errors and data cleaning performed.

Demographic characteristics of participants at baseline were compared using the Fisher exact test, Pearson chi-square test, and ANOVA. Descriptive tests were used to analyze baseline EHL assessment. Testing of hypothesis, for between-group changes in EHL scores at one-month, was performed using McNemar test and Wilcoxon matched-pairs test. All data analysis was two-sided at a 5% significance level and performed using the Statistical Package for Social Sciences (SPSS 21).

## Results

### Recruitment

**Fig 2:**
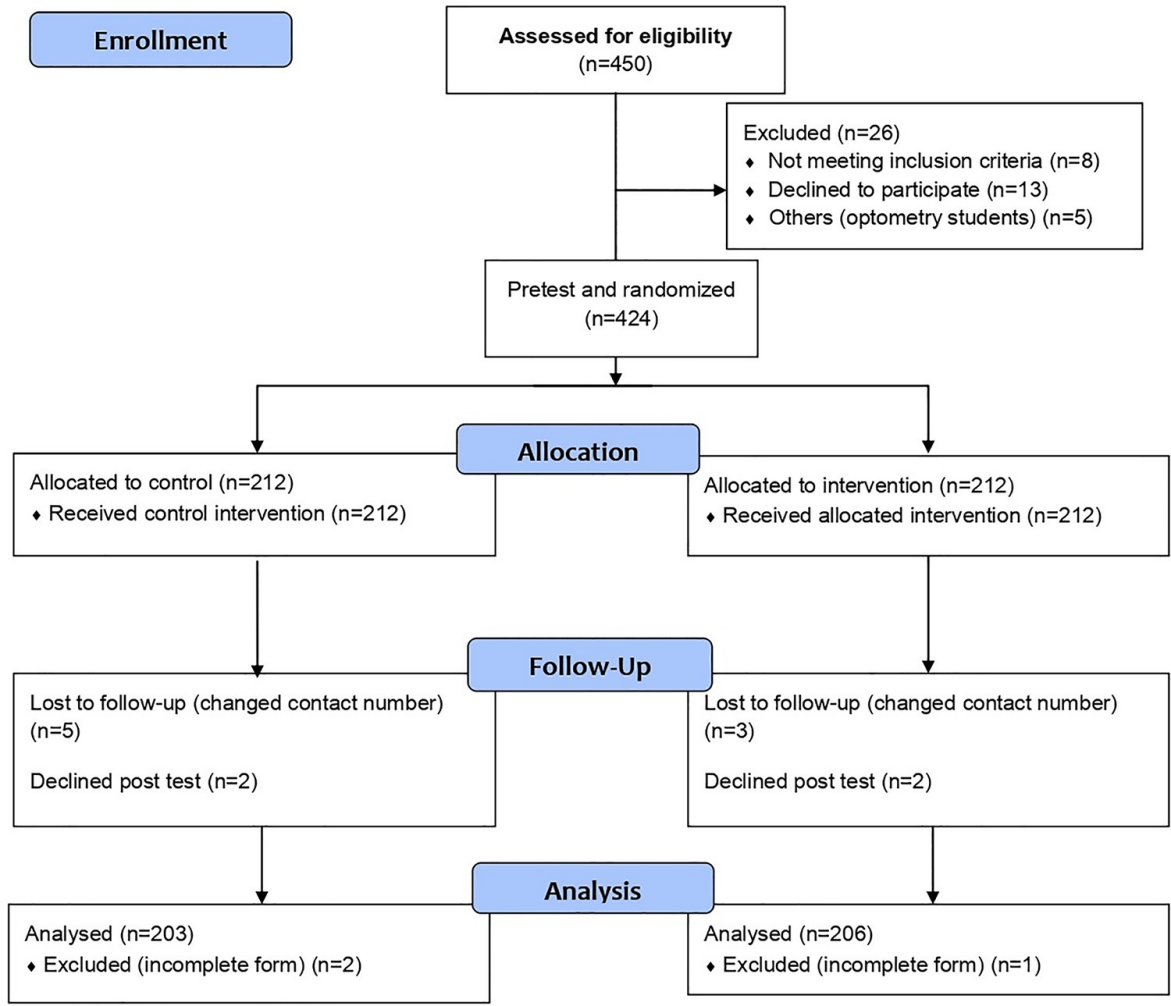
The participant flow from enrollment through analysis.

A total of 450 students were assessed for eligibility; 428 recruited, 424 randomised and 409 response analysed. Using the 1:1 allocation approach, 424 were randomized into intervention and control groups equally (n = 212). After the post-intervention assessment, only 409 responses (203 control group and 206 intervention group) were eligible for the statistical analysis (response rate, 95.6%) The participant flow from enrollment through analysis is depicted in **Fig 2**.

### Baseline characteristics

**Fig 3:**
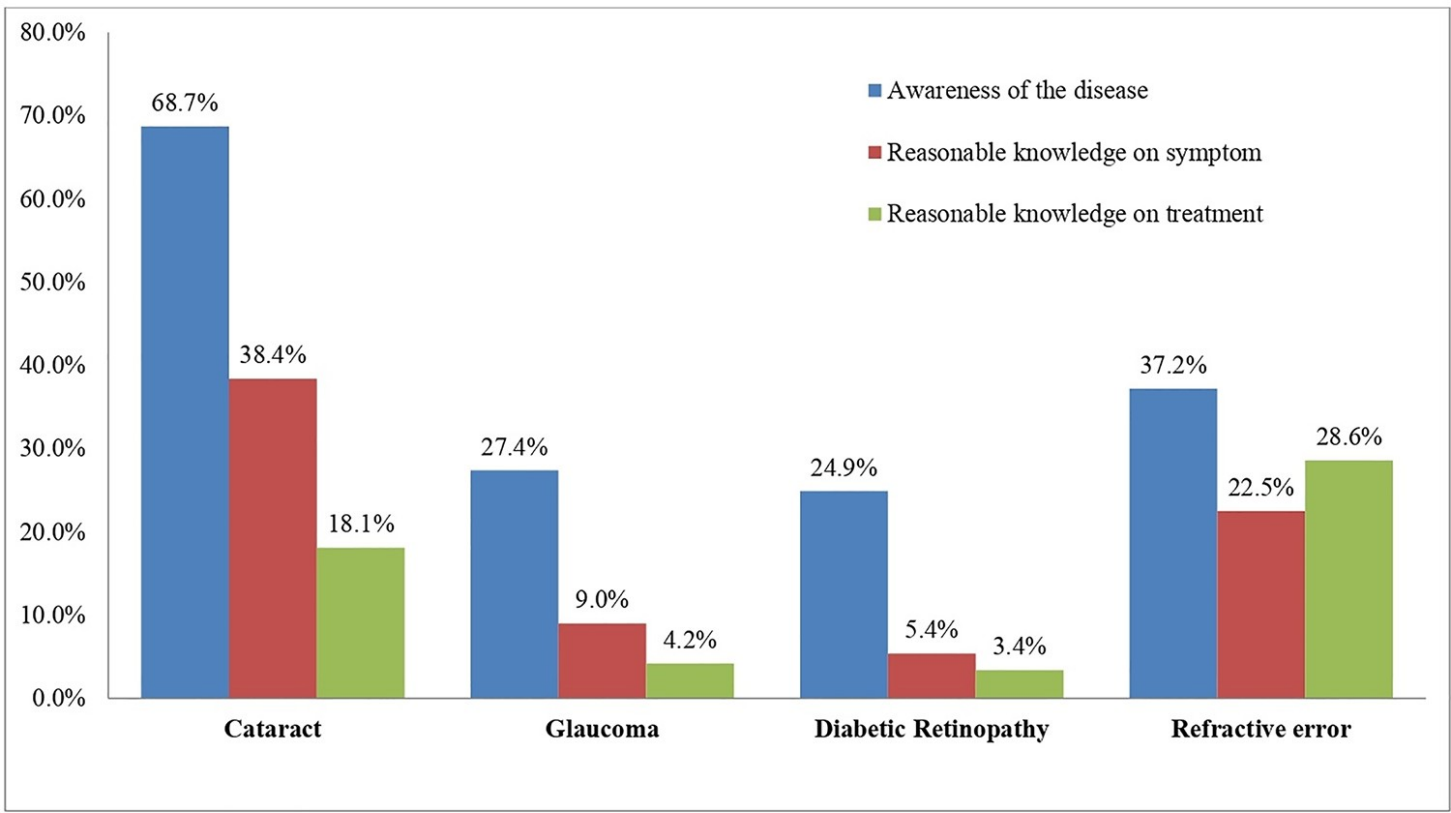
The baseline EHL scores of the study participants.

The mean age of the participants (49.4% males and 50.6% females) was 19.9±1.68 (range 18–26) years. At the baseline, the demographic characteristics were similar between the two groups (for all, P <0.05, CI 95%). Notably, there were more undergraduate students and of Hindu ethnicity. There was no significant correlation between the EHL scores and demographic profile (all p<0.05, CI 95%). The baseline demographic characteristics of the study participants are summarised in Table 1. The baseline EHL scores of the study participants are summarized in **Fig 3**.

**Table 1:** Homogeneity of demographic characteristics of the participants at the baseline.

Variables		Control (1) n = 203	Intervention (2) n = 206	Total (n = 409)	P value
**Age (years± SD)**		19.77±1.58	20.01±1.77	19.9±1.68	0.155*
**Gender, n (%)**					
	Male	99 (49.0)	103 (51.0)	202	0.843^†^
	Female	104 (50.2)	103 (49.8)	207	
**Religion, n (%)**					
	Hindu	183 (50.3)	181 (49.7)	364	0.701***
	Muslim	7 (50.0)	7 (50.0)	14	
	Christian	8 (50.0)	8 (50.0)	16	
	Sikh	4 (40.0)	6 (60.0)	10	
	Others	1 (25.0)	4 (75.0)	5	
**Course, n (%)**					
	Undergraduate	182 (50.1)	181(49.9)	363	0.640^†^
	Postgraduate	21 (45.7)	25 (54.3)	46	
**Study Program, n (%)**					
	Arts	36 (53.7)	31(46.3)	67	0.056^‡^
	Business studies	53 (57.6)	39 (42.4)	92	
	Engineering	45 (39.5)	69 (60.5)	114	
	Science	69 (50.7)	67 (49.7)	136	
**Year of study, n (%)**					
	First	71 (56.8)	54 (43.2)	125	0.059^‡^
	Second	74 (46.3)	86 (53.7)	160	
	Third	49 (51.6)	46 (48.4)	95	
	Fourth	9 (31.0)	20 (69.0)	29	
**History of eye disease? n (%)**					
	No	182 (49.3)	187 (50.7)	369	0.738^‡^
	Refractive error	18 (54.5)	15 (45.5)	33	
	Diabetic retinopathy	2 (66.7)	1 (33.3)	3	
	Glaucoma	1 (33.3)	2 (66.7)	3	
	Cataract	0 (0.0)	1 (100)	1	
**History of eye disease in the family? n (%)**					
	No	142 (52.2)	130(47.8)	272	0.253^‡^
	Cataract	13 (38.2)	21 (61.8)	34	
	Glaucoma	2 (100)	0 (0.0)	2	
	Diabetic Retinopathy	5 (62.5)	3 (37.5)	8	
	Refractive error	11 (50.0)	11 (50.0)	22	
	Don’t Know	30 (42.2)	41 (57.8)	71	

SD, standard deviation.

*One way ANOVA between mean of groups.

^†^ Fisher’s exact test between group differences.

^‡^ Pearson Chi-square test between group differences.

### Primary outcome

A statistical comparison found that both groups demonstrated better EHL (awareness and knowledge) scores at post-test compared to baseline assessment. **Table 2** shows the comparison of EHL score in the two groups, pre and post text message link intervention. The intervention group had a higher EHL score (p≤0.001, CI 95% for all) after mobile health intervention (text message link) than of baseline. This finding supported hypothesis 1.

**Table 2:** Changes in EHL (awareness and knowledge) scores between the pre-and post-test assessment between the two groups.

Disease	Outcome measure	Control Group n(%)			Intervention Group n(%)		
		Pre-test	Post-test	*p-value*	Pre-test	Post-test	*p-value*
**Cataract**	Awareness	142 (67.0)	142 (69.5)	0.89	139 (65.6)	153 (74.27)	<0.000
	RKS	86 (40.6)	88 (42.0)	0.50	71 (33.5)	82 (39.8)	0.001
	RKT	40 (18.9)	48 (23.6)	0.01	34 (16.0)	48 (23.3)	<0.000
**Glaucoma**	Awareness	48 (22.6)	50 (24.6)	0.50	64 (30.2)	95 (46.1)	<0.000
	RKS	19 (9.0)	19 (9.4)	0.97	18 (8.5)	42 (20.3)	<0.000
	RKT	11 (5.2)	15 (7.4)	0.125	6 (2.8)	24 (11.6)	<0.000
**Diabetic retinopathy**	Awareness	49 (23.1)	55 (27.1)	.210	53 (25)	85 (41.2)	<0.000
	RKS	12 (5.6)	17 (8.4)	0.063	10 (4.7)	31 (15.4)	<0.000
	RKT	8 (3.8)	12 (5.9)	0.125	6 (2.8)	28 (13.6)	<0.000
**Refractive error**	Awareness	77 (36.3)	90 (44.3)	<0.000	75 (35.4)	115 (55.8)	<0.000
	RKS	49 (23.1)	56 (27.6)	0.016	43 (20.3)	67 (33.0)	<0.000
	RKT	54 (25.5)	65 (32.0)	0.080	63 (29.7)	98 (47.5)	0.001

RKS, reasonable knowledge of symptoms; RKT, reasonable knowledge on treatment.

The changes in EHL scores between the pre-and post-test assessment between the two groups is shown in **Table 3**. At one-month post-test, the intervention group had a higher EHL score after intervention than that of the control group (p≤0.001, CI 95% for all). Thus, hypothesis 2 was supported.

**Table 3:** The changes in EHL scores between the pre-and post-test assessment between the two groups.

Ocular Condition	Control Group n(%)				Intervention Group n(%)				(Time x Group)
	Baseline EHL score	Post-test EHL score	Mean difference (95% CI)	p-value	Baseline EHL score	Post-test EHL score	Mean difference (95% CI)	p-value	p-value
**Cataract**	1.34±1.05	1.35±1.05	0.010 (0.00 to 0.02)	0.16	1.18±1.03	1.23±1.08	0.04 (0.02 to 0.07)	0.003	0.001
**Glaucoma**	0.38±0.78	0.39±0.76	0.010 (0.00 to 0.02)	0.16	0.46±0.71	0.58±0.71	0.12 (0.07 to 0.16)	0.000	0.001
**Diabetic Retinopathy**	0.36±0.76	0.37±0.77	0.010 (0.00 to 0.02)	0.16	0.32±0.61	0.46±0.64	0.14 (0.09 to 0.18)	0.000	0.001
**Refractive Error**	0.63±0.95	0.65±0.95	0.15 (0.00 to0.32)	0.08	0.63±0.95	0.7±0.92	0.10 (0.06 to 0.14)	0.000	0.003

EHL, eye health literacy; CI, confidence interval.

### Secondary outcome

In the intervention group, 87.8% (181 of 206) participants responded they opened the hyperlink in the text message, while 83.4% (172 of 206) participants found it useful. Each text message costs us an average of INR 0.50; the total cost was INR 818 (USD 11.5).

## Discussion

Finding cost-effective methods for eye health promotion is an eye health priority, particularly in the LMICs. There is firm evidence that mobile phone messages can successfully promote healthcare, improve medication adherence, and change health behaviour [21–23]. This study has added fresh evidence supporting mobile health interventions (mobile text link) could effectively promote eye-health; the first of its kind to the best of investigators knowledge. Evidence of any effective eye health promotion methods that could benefit public health planning and advocacy is a crucial part of VISION 2020: Right to Sight [24]. With ubiquitous access and an increasingly popular communication platform even in the LMICs, text message tool is a well-established intervention for public health [25]. Recognizing that the number of characters in text messaging limits adequate dissemination of health information, this study aimed to capture if text message links could help mhealth interventions reach their full potential as a health advocacy strategy.

The baseline assessment suggests that the awareness of priority eye conditions was relatively poor amongst the study population; cataracts 68.7%, glaucoma 27.4%, diabetic retinopathy 24.9%, and refractive error 37.2%. Nevertheless, it is worthy to note that these findings align with the previous studies conducted in Asia [26, 27]. The knowledge of these common eye conditions is also poor, and correlates well with literature. A Low EHL score could mean inadequate or ineffective eye health advocacy programs in the community.

With the advancements in technologies over the past decade, a revolution has been occurring in health promotion [28]. In this study, the most notable finding was that, in the intervention group, the ESL scores after the intervention showed significant improvements for all four ocular conditions (p>0.05, CI 95%, for all). As hypothesized, EHL scores were significantly higher for intervention than that of the control group at one month. Though no studies were available to compare our findings on eye health, the results were consonant with the observations on other health care interventions [14, 15]. Text message link was also employed effectively as an online survey tool for longitudinal data collection [29]. The study hypotheses were supported by the results, establishing that text message link intervention could effectively promote EHL.

The improvement in EHL in the group receiving text message link intervention could be attributed to several reasons. The mhealth technologies are portable and online materials can be easily accessed at individual’s convenience with an internet connection. It allows the intervention to claim an individual’s attention at the most convenient time by allowing temporal synchronization of the intervention delivery [30]. In 2019, more than half of the global population (4.13 billion) was connected to the internet via mobile phones [31]. The text message link intervention offers an additional advantage of not requiring to send multiple messages to disseminate a considerable amount of information. As more people use mobile phones throughout the day for various tasks, it is less likely to miss a text message prompt [32].

Although mobile health interventions are presently not utilized in public health for eye care, it was proven cost-effective as reminder programs [30]. Literature suggests a tremendous potential for text message links to positively affect public health intervention, particularly in the LMICs [33]. The cost-effectiveness of the method employed in this study can be established by the cost incurred (less than USD 12) to disseminate advocacy material through a hyperlink to the study population. This study provides shreds of evidence that sending text messages linked to a reliable website could be an effective medium for promoting EHL. While this study used English language-based text messages and a link to a English language website, interventions in vernacular languages could be even more cost-effective.

The COVID 19 pandemic has attacked the health system; ensuring access to health services is the cornerstone of successful health response [34]. During the COVID-19 pandemic more than ever, identifying and implementing effective health advocacy strategies is critical to enabling a better global response. A text message link is a tool to disseminate and reinforce information for eye health promotion effectively. Given its effectiveness, the text message link intervention could be considered a tool for equalizing access to information to address health disparities in minority populations.

### Limitation

While evidence in this study is largely in favour of text message link interventions, this study was not without limitations. The study cohort comprised the same university students, so the possibility of diffusion could not be eliminated. The generalization of the findings is also limited to university students. In the future, multicenter randomised controlled trials (RCTs) could address issues on how the program can be made more cost-effective. Registration of the study as a randomized controlled trial (RCT) was not possible within the stipulated time.

## Conclusion

The evaluation of mobile health intervention in this study provided evidence that text message links could improve EHL. The substantially low baseline EHL scores amongst educated individuals are of concern and call for the adoption of effective strategies for eye health promotion. Significantly higher EHL scores in the intervention group establish that text message links help promote eye health. Text message link intervention is also cost effective, and it could be used for promoting eye health, particularly by the LMICs during the current coronavirus pandemic.

## Supporting information

S1 File Questionnaire used for the study. (DOCX)

S1 Table Participant’s responses on knowledge of priority eye disease during the baseline assessment. (DOCX)

S1 Dataset (XLS)

## References

[1] GBD, Vision Loss Expert Group of the Global Burden of Disease Study. Causes of blindness and vision impairment in 2020 and trends over 30 years, and prevalence of avoidable blindness in relation to VISION 2020: the Right to Sight: an analysis for the Global Burden of Disease Study. Lancet Glob Health. 2020;2020(December 1):S2214–S109X. doi: 10.1016/S2214-109X(20)30489-7 . 33275949 PMC7820391

[2] Jaggernath J, Øverland L, Ramson P, Kovai V, Chan VF, Naidoo KS. Poverty and eye health. Health. 2014;06(14):1849–1860.

[3] Eckert KA, Carter MJ, Lansingh VC, Wilson DA, Furtado JM, Frick KD, et al. A Simple Method for Estimating the Economic Cost of Productivity Loss Due to Blindness and Moderate to Severe Visual Impairment. Ophthalmic Epidemiol. 2015;22(5):349–55. doi: 10.3109/09286586.2015.1066394 26395661

[4] GBD 2019 Blindness and Vision Impairment Collaborators; Vision Loss Expert Group of the Global Burden of Disease Study. Trends in prevalence of blindness and distance and near vision impairment over 30 years: an analysis for the Global Burden of Disease Study. Lancet Glob Health. 2020 Dec 1:S2214-109X(20)30425-3. doi: 10.1016/S2214-109X(20)30425-3 33275950 PMC7820390

[5] Flaxman SR, Bourne RRA, Resnikoff S, Ackland P, Braithwaite T, Cicinelli MV, et al.; Vision Loss Expert Group of the Global Burden of Disease Study. Global causes of blindness and distance vision impairment 1990–2020: a systematic review and meta-analysis. Lancet Glob Health. 2017 Dec;5(12):e1221–e1234. doi: 10.1016/S2214-109X(17)30393-5 29032195

[6] Thapa R, Bajimaya S, Paudyal G, Khanal S, Tan S, Thapa SS, et al. Population awareness of diabetic eye disease and age related macular degeneration in Nepal: the Bhaktapur Retina Study. BMC Ophthalmol. 2015 Dec 29;15:188. doi: 10.1186/s12886-015-0175-z 26714483 PMC4696239

[7] Islam FM, Chakrabarti R, Islam SZ, Finger RP, Critchley C. Factors Associated with Awareness, Attitudes and Practices Regarding Common Eye Diseases in the General Population in a Rural District in Bangladesh: The BangladeshPopulation-based Diabetes and Eye Study (BPDES). PLoS One. 2015 Jul 22;10(7):e0133043. doi: 10.1371/journal.pone.0133043 26200458 PMC4511641

[8] Aboobaker S, Courtright P. Barriers to Cataract Surgery in Africa: A Systematic Review. Middle East African Journal of Ophthalmology. 2016;23(1):145–149. doi: 10.4103/0974-9233.164615 26957856 PMC4759895

[9] Marmamula S, Khanna RC, Shekhar K, Rao GN. A population-based cross-sectional study of barriers to uptake of eye care services in South India: the Rapid Assessment of Visual Impairment (RAVI) project. BMJ Open. 2014 Jun 12;4(6):e005125. doi: 10.1136/bmjopen-2014-005125 24928590 PMC4067889

[10] Katibeh M, Blanchet K, Akbarian S, Hosseini S, Ahmadieh H, Burton MJ. Planning eye health services in Varamin district, Iran: a cross-sectional study. BMC Health Serv Res. 2015 Apr 3;15:140. doi: 10.1186/s12913-015-0797-0 26021828 PMC4517388

[11] Lefebvre C. Integrating cell phones and mobile technologies into public healthpractice: a social marketing perspective. Health Promot Pract. 2009Oct;10(4):490–494. 10.1177/1524839909342849 19809002

[12] Hall AK, Cole-Lewis H, Bernhardt JM. Mobile text messaging for health: a systematic review of reviews. Annu Rev Public Health. 2015;36:393–415. doi: 10.1146/annurev-publhealth-031914-122855 25785892 PMC4406229

[13] Mitchell SJ, Godoy L, Shabazz K, Horn IB. Internet and mobile technology useamong urban African American parents: survey study of a clinical population. JMed Internet Res. 2014 Jan 13;16(1) 10.2196/jmir.2673 PMC3906691 24418967

[14] Hall CS, Fottrell E, Wilkinson S, Byass P. Assessing the impact of mHealth interventions in low- and middle-income countries—what has been shown to work? Glob Health Action. 2014 Oct 27;7:25606. doi: 10.3402/gha.v7.25606 25361730 PMC4216389

[15] Zurovac D, Sudoi RK, Akhwale WS, Ndiritu M, Hamer DH, Rowe AK, et al. The effect of mobile phone text-message reminders on Kenyan health workers’ adherence to malaria treatment guidelines: a cluster randomised trial. Lancet. 2011 Aug 27;378(9793):795–803. doi: 10.1016/S0140-6736(11)60783-6 21820166 PMC3163847

[16] Arambepola C, Ricci-Cabello I, Manikavasagam P, Roberts N, French DP, Farmer A. The Impact of Automated Brief Messages Promoting Lifestyle Changes Delivered Via Mobile Devices to People with Type 2 Diabetes: A Systematic Literature Review and Meta-Analysis of Controlled Trials. J Med Internet Res. 2016 Apr 19;18(4):e86. doi: 10.2196/jmir.5425 ; PMCID: PMC4873307. 27095386 PMC4873307

[17] Hubley J, Gilbert C. Eye health promotion and the prevention of blindness in developing countries: critical issues. Br J Ophthalmo.2006;90(3):279–284. doi: 10.1136/bjo.2005.078451 16488944 PMC1856969

[18] Fedele DA, Cushing CC, Fritz A, Amaro CM, Ortega A. Mobile Health Interventions for Improving Health Outcomes in Youth: A Meta-analysis. JAMA Pediatr. 2017 May 1;171(5):461–469. doi: 10.1001/jamapediatrics.2017.0042 Erratum in: JAMA Pediatr. 2017 May 1;171(5):495. Erratum in: JAMA Pediatr. 2018 Mar 1;172(3):302. ; PMCID: PMC6037338.] 28319239 PMC6037338

[19] Kampmeijer R, Pavlova M, Tambor M, Golinowska S, Groot W. The use of e-health and m-health tools in health promotion and primary prevention among older adults: a systematic literature review. BMC Health Serv Res. 2016 Sep 5;16 Suppl 5(Suppl 5):290. doi: 10.1186/s12913-016-1522-3 ; PMCID: PMC5016733. 27608677 PMC5016733

[20] Källander K, Tibenderana JK, Akpogheneta OJ, Strachan DL, Hill Z, ten Asbroek AH, et al. Mobile health (mHealth) approaches and lessons for increased performance and retention of community health workers in low- and middle-income countries: a review. J Med Internet Res. 2013 Jan 25;15(1):e17. doi: 10.2196/jmir.2130 ; PMCID: PMC3636306. 23353680 PMC3636306

[21] Hall AK, Cole-Lewis H, Bernhardt JM. Mobile text messaging for health: a systematic review of reviews. Annu Rev Public Health. 2015;36:393–415. doi: 10.1146/annurev-publhealth-031914-122855 25785892 PMC4406229

[22] Armanasco AA, Miller YD, Fjeldsoe BS, Marshall AL. Preventive Health Behavior Change Text Message Interventions: A Meta-analysis. Am J Prev Med. 2017 Mar;52(3):391–402. doi: 10.1016/j.amepre.2016.10.042 Epub 2017 Jan 7. . 28073656

[23] Thakkar J, Kurup R, Laba TL, Santo K, Thiagalingam A, Rodgers A, et al. Mobile Telephone Text Messaging for Medication Adherence in Chronic Disease: A Meta-analysis. JAMA Intern Med. 2016 Mar;176(3):340–9. doi: 10.1001/jamainternmed.2015.7667 . 26831740

[24] Advocacy for eye health. Community Eye Health. 2007;20(64):57–59. 18330435 PMC2206315

[25] Smith DM, Duque L, Huffman JC, Healy BC, Celano CM. Text Message Interventions for Physical Activity: A Systematic Review and Meta-Analysis. Am J Prev Med. 2020 Jan;58(1):142–151. doi: 10.1016/j.amepre.2019.08.014 Epub 2019 Nov 21. ; PMCID: PMC6956854. 31759805 PMC6956854

[26] Dandona R, Dandano L, John RK. et al. Awareness of eye diseases in an urban population in southern India. BullWorld Health Organ. 2001; 79: 96–102. PMC2566357 11242828

[27] Lau JT, Lee V, Fan D, Lau M, Michon J. Knowledge about cataract, glaucoma, and age related macular degeneration in the Hong Kong Chinese population. Br J Ophthalmol. 2002 Oct;86(10):1080–4. doi: 10.1136/bjo.86.10.1080 ; PMCID: PMC1771305. 12234882 PMC1771305

[28] Sahu M, Grover A, Joshi A. Role of mobile phone technology in health education in Asian and African countries: a systematic review. Int J Electron Healthc. 2014;7(4):269–86. doi: 10.1504/IJEH.2014.064327 25161104

[29] Friedlander EB, Soon R, Salcedo J, Tschann M, Fontanilla T, Kaneshiro B. Text message link to online survey: A new highly effective method of longitudinal data collection. Contraception. 2020 Apr;101(4):244–248. doi: 10.1016/j.contraception.2019.11.008 Epub 2019 Dec 27. 31884078

[30] Free C, Phillips G, Watson L, Galli L, Felix L, Edwards P, et al. The effectiveness of mobile-health technologies to improve health care service delivery processes: a systematic review and meta-analysis. PLoS Med. 2013;10(1):e1001363. doi: 10.1371/journal.pmed.1001363 23458994 PMC3566926

[31] Statista. Internet usage worldwide–statistics & facts. Available from: https://www.statista.com/topics/1145/internet-usage-worldwide/ [accessed on 29th December 2020]

[32] Zurovac D, Larson BA, Sudoi RK, Snow RW. Costs and Cost-Effectiveness of a Mobile Phone Text-Message Reminder Programmes to Improve Health Workers’ Adherence to Malaria Guidelines in Kenya. Smith TA, ed. PLoS ONE. 2012;7(12):e52045. doi: 10.1371/journal.pone.0052045 23272206 PMC3525566

[33] Consulting VW. mHealth for Development: The Opportunity of Mobile Technology for Healthcare in the Developing World. Washington D.C. and Berkshire, UK; 2009.

[34] WHO. Attacks on health care in the context of COVID-19. Available from:https://www.who.int/news-room/feature-stories/detail/attacks-on-health-care-in-the-context-of-covid-19.[accessed on 30th November 2020]

