# Supplementary material for: Mobile health intervention for promotion of eye health literacy
Source: PLOS Glob Public Health. 2021 Oct 13;1(10):e0000025. doi: 10.1371/journal.pgph.0000025 (PMC10021255; doi:10.1371/journal.pgph.0000025)
Supplement: S1 File — (DOCX) [file pgph.0000025.s001.docx]

**Please answer the following questions.**

**A. Cataract**

A1. Have you heard of the disease ? Yes No

A2. What are the common symptoms?

A3. Please name the appropriate treatment.

**B. Glaucoma**

B1. Have you heard of the disease ? Yes No

B2. What are the common symptoms?

B3. Please name the appropriate treatment.

C**. Diabetic Retinopathy**

C1. Have you heard of the disease ? Yes No

C2. What are the common symptoms?

C3. Please name the appropriate treatment.

**D. Refractive Errors**

D1. Have you heard of the disease ? Yes No

D2. What are the common symptoms?

D3. Please name the appropriate treatment.

**E1.** Did you open the hyperlink sent to you as text message? Yes No

**E2.** If YES, was the hyperlink useful? Yes No

***Footnote:*** *Questions E1 and E2 were not included in the pre-test.*
