# Supplementary material for: Mobile health intervention for promotion of eye health literacy
Source: PLOS Glob Public Health. 2021 Oct 13;1(10):e0000025. doi: 10.1371/journal.pgph.0000025 (PMC10021255; doi:10.1371/journal.pgph.0000025)
Supplement: S1 Table — (DOCX) [file pgph.0000025.s002.docx]

**S2 Table.**  Participant’s responses on knowlege of eye diseases received during the pre-test.

| 1. What are the symptoms? | | | |
| --- | --- | --- | --- |
| **Cataract** | **Glaucoma** | **Diabetic Retinopathy** | **Refractive Error** |
| Black spot on the retina | Blurr vision and pain * | Blur vision * | Blurred vision * |
| Blindness * | Increase pressure in eye * | Body weakness | Blindness |
| Blur/hazy vision * | Blindness * | Colour blindness | Colour defect |
| Cloudy cornea | Optic nerve damage * | Increase sugar level * | Nearsighted and far sighted * |
| Pain and redness | Redness | Retina problem * | *Low Vision ** ^¥^ |
| White lens or pupil * | White cornea | *New blood vessels in retina* * ^¥^ |  |
| *Low vision ** ^¥^ | *Optic nerve disease ** | *Bleeding in the retina ** ^¥^ |  |
| *Clouding of lens of eye ** ^¥^ |  |  |  |
| **2. How is it treated?** | | | |
| **Cataract** | **Glaucoma** | **Diabetic Retinopathy** | **Refractive Error** |
| Surgery * | Laser surgery * | Diet control * | Green vegetable |
| Drugs | Lenses | Drugs* | LASIK * |
| Laser treatment | Surgery * | Laser * | Refractive surgery * |
| Nutrition | Surgery and eyedrops * | Surgery * | Spectacle * |
| Spectacle | *Medicines **^¥^ | Spectacle | Contact lens * |
| Removal of eye lens and replacement with artificial * |  | *Decrease blood sugar ** ^¥^ |  |
| *considered as “correct response” and as having reasonable knowledge  ^¥^ new responses received during post-test | | | |
