# Supplementary material for: Mobile health intervention for promotion of eye health literacy
Source: PLOS Glob Public Health. 2021 Oct 13;1(10):e0000025. doi: 10.1371/journal.pgph.0000025 (PMC10021255; doi:10.1371/journal.pgph.0000025)
Supplement: Attachment — Submitted filename: Response to reviewers.docx [file pgph.0000025.s004.docx]

**Response to reviewers**

| **Sl no** | **Reviewer’s comments** | **Authors response** |
| --- | --- | --- |
| 1 | Reviewer #1: The authors present a well written article documenting the use of text messaging as a means of improving eye health literacy in a low middle-income country. They clearly demonstrate an increased awareness of eye related issues in the participants in the intervention group.  However, there are certain questions that need to be answered. | Thank you for your kind review and comments. The authors have tried our best to address all concerns raised. |
| 2 | Did the participants know they were in an interventional study (although they might not know what the intervention was)? | YES. While signing the informed consent, participants were informed that they were in an intervention study. However, they didn’t know what the intervention was. |
| 3 | Did they know they would receive a Free Eye Exam at the time of enrollment? | YES. While inviting for the study, participants were informed that they would receive a free eye exam if they participated throughout the study. This was done to encourage more participants and reduce dropouts. |
| 4 | Add in the key for the demographic data that needs to be added just for clarity. | Keys are added at the bottom of the table |
| 5 | As the authors pointed out they selected a very defined population of young, presumably English speaking, college students. Such a population does not necessarily reflect the target population. | The study population may not necessarily reflect the target population. This is a limitation and mentioned in the ‘Limitation’ section. |
| 6 | Another issue is the language used. The authors used an English language-based text message and a link to a English language website. Would this be an issue when dealing with a diverse population such as in India, especially from a public health perspective? Many of the target populations may not be fluent/comfortable with English.  Any thoughts on the use of vernacular languages?? | As of January 2021, the internet penetration in India was 45% (624 million users). From a public health perspective, interventions could be done with vernacular languages. While this study used English based text, using local websites (in local languages) could be even more effective. |
| 7 | Did any of the participants or their family have any eye issues other than those listed in the paper? | This was not asked and will not be able to comment on it. |
| 8 | Are smartphones as a means of public health measures feasible in the rural parts of a country? | 40% of the smartphone users are from rural India (<https://indianexpress.com/article/technology/tech-news-technology/icea-report-83-crore-smartphone-users-by-2022-6499952/>). So smart phones could be a cost effective measure. |
| 9 | I believe the authors touched up on this but is it possible that some students talked to each other, perhaps two people, one of whom got the link and one who did not? | The possibility of diffusion (talking to each other about the link) could not be ruled out. This is mentioned in the limitation of the study. |
| 10 | Can the increased EHL scores be due to the general increased interest since they were participating in clinical trials or the intervention itself? | While all participants were aware that they were in an interventional study, only those in the intervention group showed significant increase in EHL scores. This could mean that there may not have been an increased interest. However, this could not be ruled out. |
| 11 | Were there any differences in sub-group analysis, e.g. within the intervention group did the Science majors do better than Arts Majors? | There were no significant differences between subgroups. This is now mentioned in the results. |
